# Risk Factors Associated with Multiple Sclerosis: A Case-Control Study in Damascus, Syria

**DOI:** 10.1155/2021/8147451

**Published:** 2021-06-01

**Authors:** Maher Taan, Farah Al Ahmad, Mohammad Karim Ercksousi, Ghassan Hamza

**Affiliations:** Faculty of Medicine, Damascus University, Damascus, Syria

## Abstract

**Objectives:**

To assess the probable risk factors associated with Multiple sclerosis among Syrian patients in the city of Damascus.

**Method:**

In a case-control study conducted from May to September 2020, 140 MS patients and 140 healthy controls were selected from two main hospitals in Damascus. Data regarding risk factors associated with MS was collected via a structured questionnaire and complementary laboratory tests. The statistical analysis was carried out by the SPSS Statistical Software Version 26.

**Results:**

Factors such as smoking, family history of MS, migraine, and vitamin D deficiency were associated with a higher risk of developing MS: Smoking (OR = 2.275 95% CI (1.348-3.841) *P* = 0.002). Family history of MS (OR = 3.970 95% CI (1.807-8.719) *P* ≤ 0.001). Migraine (OR = 3.011 95% CI (1.345-6.741) *P* = 0.005). Vitamin D deficiency (OR = 4.778 95% CI (2.863-7.972) *P* ≤ 0.001). However, factors such as diabetes, hypertension, a surgical history of appendectomy, tonsillectomy, and being the first-born in a family were statistically irrelevant: Diabetes (OR = 0.652 95% CI (0.226-1.882) *P* = 0.426). Hypertension (OR = 1.445 95% CI (0.724-2.885) *P* = 0.295) Appendectomy (OR = 1.269 95% CI (0.486-3.317) *P* = 0.626) Tonsillectomy (OR = 1.280 95% CI (0.576-2.843) *P* = 0.544). First-born Child (OR = 0.933 95% CI (0.558-1.562) *P* = 0.793).

**Conclusion:**

Our study suggests that smoking, vitamin D deficiency, family history of MS, and migraine are probable risk factors for multiple sclerosis. Therefore, engaging in outdoor activities and maintaining a healthy diet—for females in particular—is highly recommended.

## 1. Introduction

Multiple sclerosis is an inflammatory, autoimmune demyelinating disease of the central nervous system affecting more than 2 million people around the world- with an estimated overall prevalence of 51.52/100000 in the Middle East alone [1]. Previous studies have shown that different genetic, immunological, and environmental factors play a role in the pathogenesis of this disease. However, the underlying cause and mechanisms behind MS remain unclear. The epidemiology of MS is changing, despite the increasing awareness raised against chronic diseases and the accessibility of new diagnostic procedures. Recent data has reflected an increase in the prevalence of the disease across Europe, Latin America, and the Mediterranean Basin [2]. Factors including geographic latitude, amount of daylight exposure, viruses, smoking, and nutritional habits—among others—were linked with MS in several studies [3–6]. In addition, clinically significant depressive symptoms were found in 41.8% of MS subjects in a study conducted by Chwastiak et al. [7]. The study demonstrated that as a disease, MS tends to be mentally draining, not only for the patients but also for their families and the greater community as well. Our study aims to evaluate the relationship between probable risk factors and MS by using a case-control study design in Syria. Taking into consideration that few studies have addressed this topic in our region, it was necessary to investigate the environmental triggers of MS in order to achieve a better understanding of how this disease functions.

## 2. Material and Methods

We selected a total number of 140 MS patients and 140 healthy controls who were matched for gender and age (±1 year) from two main hospitals in Damascus: Al-Assad University Hospital and Ibn Al-Nafees Hospital. Our inclusion criteria for MS cases consisted of patients who were able to fulfill the McDonald 2017 criteria had been diagnosed or admitted in either of the hospitals within the past year (2019-2020) and were 18 years or older. Exclusion criteria for MS cases consisted of patients who were not able to fulfill the McDonald 2017 criteria were less than 18 years old or were unable to understand the questionnaire and provide detailed answers. The total number of cases that matched our criteria reached approximately 250 patients. However, to avoid any sampling bias, the 250 patients were divided into two groups based on gender, from which we then randomly selected the subjects who would be included in our study until we reduced our sample size to 140 cases. While doing this, we made sure to take into consideration the importance of maintaining the same female to male ratio of the initial population. For the controls, we approached the healthy companions of the patients in other hospital departments—excluding the Neurology Department—while following our exclusion criteria for the controls (subjects with a history of MS, under 18 years of age, or who were unable to understand the questionnaire and provide detailed answers).

For this study, we adopted a structured questionnaire which included several questions about demographics, neurological symptoms, family history, smoking, vitamin D deficiency, weight, height, history of chronic illnesses, and history of surgical procedures. We assessed our questionnaire's validity by conducting several pilot surveys. In addition, we presented the questionnaire to different neurologists to consider their opinions and feedback. To evaluate our questionnaire's reliability, we used a test-retest method, administering the same questionnaire to the same group of people at different times and, thereafter, assessing the degree of similarity between the two sessions of the questionnaire. Through the questionnaire, we collected data from both the cases and the controls regarding the following factors: smoking, family history of MS, tonsillectomy, and appendectomy. For the data regarding vitamin D deficiency, migraine, hypertension, and diabetes, we revised the patients' medical history folders in the hospitals' archives for every patient who was included in the study.

From these folders, the necessary data was collected after assessing the laboratory and clinical tests that had been performed on the respective patients after their admission to the hospital. If there was any lack or insufficient information in the patients' medical archives, the proper clinical and/or laboratory tests were performed on the subjects. As for controls, we conducted all the clinical and/or laboratory tests needed for the study. After confirmation of the protocol by the Ethics Committee of Damascus University Faculty of Medicine, all data was collected carefully under close supervision to address any inquiries and to assess whether any question was left unanswered.  Table 1 shows a detailed explanation of each variable composing the Inclusion Criteria that was used in the study.

To reach the goal of our study, a univariate analysis method was implemented, which consisted of assessing the relationship between each factor individually, and the risk of developing MS from it. Frequency percentage values were reported to demonstrate the nominal and categorical variables, and the median value was used to describe numeric variables. The Chi-Square test and the Fisher's exact test were both used to test the association between categorical variables, and the corresponding odds ratios (OR) and its 95% confidence interval (CI) were computed for each variable. In all analytical procedures, a *p* value of ˂0.05 was considered statistically significant. The statistical analysis was carried out by the SPSS Statistical Software Version 26.

## 3. Results

A total number of 140 MS patients and 140 controls were enrolled in this study. The cases were matched with the controls for gender and age, with females outnumbering males on a 2.2 : 1 female to male ratio, and the mean age was 34 years of age for both genders. The demographic comparison between the two groups is demonstrated in Table 2.

The most common subtype of MS was relapsing-remitting (RRMS) with a percentage of 60.4%. Our results showed a potential risk in the relationship between smoking and MS (OR = 2.275 95% CI (1.348-3.841) *P* = 0.002), as well as indicated a relationship between family history of MS and the risk of having the disease (OR = 3.970 95% CI (1.807-8.719) *P* ≤ 0.001). The risk of MS was also significantly higher among those who suffered from migraine (OR = 3.011 95% CI (1.345-6.741) *P* = 0.005), and an association was found between vitamin D deficiency and MS (OR = 4.778 95% CI (2.863-7.972) *P* ≤ 0.001). Results of the comparison between the two groups are summarized in Table 3.

Findings concerning the association between diabetes and risk of MS, however, were statistically irrelevant (OR = 0.652 95% CI (0.226-1.882) *P* = 0.426). High-blood pressure did not show any statistical significance for an increased risk of MS (OR = 1.445 95% CI (0.724-2.885) *P* = 0.295), and further assessments also showed no significant difference between the two groups regarding the appendectomy and tonsillectomy surgical procedures (OR = 1.269 95% CI (0.486-3.317) *P* = 0.626) and (OR = 1.280 95% CI (0.576-2.843) *P* = 0.544), respectively. Moreover, the results for being a first-born child were also statistically insignificant (OR = 0.933 95% CI (0.558-1.562) *P* = 0.793).

## 4. Discussion

The findings in our study indicated that smoking, a family history of MS, a positive history of migraine, and suffering from vitamin D deficiency are statistically more significant in MS cases than in the controls. However, variables such as suffering from hypertension, diabetes mellitus, a surgical history of tonsillectomy, appendectomy, or being the first-born child in a family showed no relation with MS and were statistically irrelevant.

Our study displayed a 2.2 : 1 female to male ratio. A similar study conducted in Syria by Almallouhi and Almallouhi [8] stated that the female to male ratio had increased from 1.8 : 1 in 2010 to 3.4 : 1 in 2015. In the greater region, other studies also reported similar ratios. For example, a study conducted in Lebanon, Yamout et al. [9] found that females outnumbered males with a female to male ratio of 1.8 : 1.

Regarding marital status, 60% of the cases were married, 35.7% were single, and the remaining cases were either divorced or widowers. Similar results are reported by Yamout et al. [9] with two-thirds of their cases having been married, while the remaining were either single or divorced.

In our study, the most common subtype of MS was relapsing-remitting multiple sclerosis (RRMS) with 60.4%, followed by progressive-relapsing multiple sclerosis (PRMS) with 22.9%. Similarly, Daif et al. [10] found that the clinical course in their study was 60.7% RRMS and 20.2% PRMS.

The clinical symptoms for MS primarily consisted of motor symptoms (34.15%), followed by sensory symptoms (24.98%), visual symptoms (21.14%), and brainstem and cerebellar symptoms (19.7%). In a study conducted by El-Salem et al. [11], visual symptoms were more common than sensory ones.

Our study suggests that there is a positive relationship between smoking and MS. Likewise, Halawani et al. [4] concluded that smoking significantly increases the risk of MS. On the other hand, Abbasi et al. [5] showed no relation between MS risk and cigarette consumption. Through further research, however, we were able to find a study by Mitrovic et al. [12], which stated that oligodendrocytes—the myelin-producing cells—are sensitive to nitric oxide exposure. Therefore, any exposure to nitric oxide will ultimately lead to cell death by either apoptosis or necrosis. This study supports the notion that the nitrogen oxide in cigarettes may be causing degeneration, demyelination, and oligodendrocytes necrosis.

The risk of MS was also higher among people with a family history of MS, which emphasizes the fact that both genetic and environmental factors play a role in the development of MS. This finding is consistent with many MS studies such as one conducted in Saudi Arabia by Al Jumah et al. [13], which reported that approximately 21% of MS patients have had a family history of MS. Unlike other studies, however, our study did not distinguish the differences between first, second, and third-degree relatives. Each individual with a family history of MS was taken into account, regardless of their degree of relationship to the family member who had MS.

In addition, our data shows more frequency of migraine in MS patients compared with the control group. While the pathogenesis behind this relation is not clear, many studies have proven the coexistence of the two diseases. Rolak and Brown [14] showed that 21% of patients with MS had migraine (22 out of 104); other studies suggested that headaches were associated with the usage of IFN beta as a treatment for MS. La Mantia et al. [15] exhibited a higher prevalence of headaches in patients treated with IFN beta (72%), compared to patients who were treated with other agents (54%). We believe that the relationship between migraine and MS is not clear to this day. Migraine may act as a risk factor for MS in some cases, while in other cases, MS itself could be the cause of migraine. It may also be possible that the two diseases just happen to coexist with one another. Due to this uncertainty, we encourage other researchers to assess this relationship to come to a better understanding of both MS and migraine.

Vitamin D is well-known to have many immunoregulatory elements and substantially interferes with a wide range of autoimmune diseases. Goldsmith [16] suggested that vitamin D may serve as an endogenous brake to inflammation. A study by Waschbisch et al. [17] also demonstrated how vitamin D induces the expression of the inhibitory receptor ILT-3, which controls the T cell activation that is essential to prevent autoimmunity. Thus, it is suggested that vitamin D cotreatment with IFN beta could contribute beneficial effects to the management of the disease. With that being said, in our study, lower serum levels of vitamin D were found to be associated with a higher risk of MS. This finding certainly stems from the overall low daylight exposure in the cases group, which led to the deficiency. This observation was also supported by other studies including Ramagopalan et al. [18] which showed that decreased UVB exposure correlates with an increased incidence of MS.

There is no clear relationship between diabetes mellitus and MS, a result which was also reflected in our investigation of the relationship between hypertension and MS. A study by Nielsen et al. [19] claimed that patients with Type 1 diabetes were at more than a threefold increased risk for the development of MS. Whereas Abbasi et al. [5] reported that diabetes mellitus appeared to be related with a decreased risk of MS. Regarding hypertension, a study conducted by Saroufim et al. [20] stated that MS cases were 48% more likely to have ever had hypertension than the controls. These unclear results regarding diabetes and hypertension in our study may be caused by the high prevalence of these two diseases in the Syrian community, which may be due to different genetic factors as well as other behavioral habits.

A surgical history of appendectomy or tonsillectomy were also statistically irrelevant in our study. However, other studies have had mixed results while studying these factors. A meta-analysis of several case controls conducted by Lunny et al. [21] suggested that there is a small yet statistically significant increased risk for developing MS for those who have undergone an appendectomy and tonsillectomy, and who are 20 years old or younger. Another study by Broadley et al. [22] claimed that no evidence was found to suggest that tonsillectomy affects the susceptibility to multiple sclerosis. The study by Eftekharian et al. [23] had the closest results to ours—showing no significant association between appendectomy and tonsillectomy, and MS—and also suggested that if more people were included in the study groups, the results could potentially show greater significant differences.

Our study also showed no statistical evidence that being the first-born child in the family represents a risk factor for MS. However, a study by Al Wutayd et al. [6] suggested the opposite by demonstrating that being the first-born child of a family is associated with an increased risk of MS. The inconsistency in these results may be due to different sets of beliefs, as well as sociodemographic characteristics such as education levels and awareness of birth control usage (both elements which may receive more emphasis in some countries than others).

Finally, our study had potential limitations. First, the lack of previous research on MS in Syria did not provide us the ability to follow an existing theoretical framework or methodology through which we could ultimately compare our results with previous studies. We believe that this factor would have enabled us to further understand how MS characteristics have evolved throughout time in Syria. Additionally, we were hindered by severe transportation limitations at the time this article was written due to the ongoing war in Syria, and thus, we were only able to sample from two hospitals. We believe that sampling from more hospitals would have enabled us to access a larger population with a broader range and/or diversity of sociodemographic characteristics, a critical factor which could have potentially led to more accurate results.

## 5. Conclusion

Our study suggests that smoking, vitamin D deficiency, family history of MS, and migraine are probable risk factors for multiple sclerosis. Therefore, engaging in outdoor activities and maintaining a healthy diet—for females in particular—is highly recommended.

## Figures and Tables

**Table 1:** Detailed inclusion criteria for each risk factor included in the study.

Smoking	This factor was considered upon the subject's statement. Smoking cigarettes, shisha, or cigars was considered smoking, in addition to the subject being a chronic smoker (meaning that they had been smoking for a minimum of 5 months, and had developed dependence to tobacco). One-time smokers were not counted as smoking.
Family history of MS	This factor was considered upon the subject's statement and their ability to recall.
Migraine	With no specific test to diagnose migraine, this factor was considered by revising the subjects' folders in the medical archives, and each subject diagnosed with migraine by a neurologist working in the hospital or outside the hospital was considered for our study. Moreover, all subjects were asked to personally confirm our findings from their medical history.
Vitamin D deficiency	This factor was defined by a laboratory test of 25-hydroxy vitamin-D in blood, a level lower than 12 ng/ml indicated vitamin D deficiency.
Diabetes	Subjects suffering from chronic diabetes and taking medications on a daily basis to sustain their normal blood sugar levels were considered for this factor. In addition to the glucose fasting serum level test that was conducted to confirm our findings, pre-diabetes was only considered for this factor if the patient was taking medication to sustain normal sugar levels.
Hypertension	Each subject suffering from chronic hypertension and needing to take medication on a daily basis to sustain normal blood pressure was considered for this factor. Prehypertension (systolic 120-139/diastolic 80-89) was only considered if the patient was taking medications to sustain normal levels.
Appendectomy	We considered this factor upon the subject's statement and with confirmation from the surgical history in the subject's folders from the hospitals' archives.
Tonsillectomy	We considered this factor upon the subject's statement and with confirmation from the surgical history in the subject's folders from the hospitals' archives.
First-born child	We considered this factor upon the subject's statements.

**Table 2:** Detailed comparison of the demographics between the cases and the controls.

		Cases percentage	Controls percentage	*P* value
Gender	Male	30.7%	30.7%	1.000
	Female	69.3%	69.3%	
Marital status	Married	60.0%	57.1%	.627
	Single	40.0%	42.9%	
Living area	Urban	74.3%	64.3%	.070
	Rural	25.7%	35.7%	
Education level	Intermediate school or lower	40.0%	46.4%	.110
	Secondary school	7.1%	12.1%	
	University or higher	52.9%	41.4%	

**Table 3:** Detailed comparison of the result values for each risk factor between the two groups.

		95% confidence interval		
	Odds ratio value	Lower	Upper	*P* value
Smoking	2.275	1.348	3.841	.002
Family history of MS	3.970	1.807	8.719	<.001
Migraine	3.011	1.345	6.741	.005
Vitamin D deficiency	4.778	2.863	7.972	<.001
Diabetes	.652	.226	1.882	0.426
High-blood pressure	1.445	.724	2.885	.296
Appendectomy	1.269	.486	3.317	.626
Tonsillectomy	1.280	.576	2.843	.544
First-born child	0.933	.558	1.562	.793

## Data Availability

The raw data supporting the results reported in this article is available upon reasonable request by contacting the corresponding author.
